# Biological responses to disturbance from simulated deep-sea polymetallic nodule mining

**DOI:** 10.1371/journal.pone.0171750

**Published:** 2017-02-08

**Authors:** Daniel O. B. Jones, Stefanie Kaiser, Andrew K. Sweetman, Craig R. Smith, Lenaick Menot, Annemiek Vink, Dwight Trueblood, Jens Greinert, David S. M. Billett, Pedro Martinez Arbizu, Teresa Radziejewska, Ravail Singh, Baban Ingole, Tanja Stratmann, Erik Simon-Lledó, Jennifer M. Durden, Malcolm R. Clark

**Affiliations:** 1 National Oceanography Centre, University of Southampton Waterfront Campus, Southampton, United Kingdom; 2 Senckenberg am Meer, German Centre for Marine Biodiversity Research (DZMB), Wilhelmshaven, Germany; 3 The Lyell Centre for Earth and Marine Science and Technology, Heriot-Watt University, Riccarton, Edinburgh, United Kingdom; 4 Department of Oceanography, University of Hawaii at Manoa, Honolulu, Hawaii, United States of America; 5 Ifremer, Centre de Bretagne, Plouzané, France; 6 Bundesanstalt für Geowissenschaften und Rohstoffe (Federal Institute for Geosciences and Natural Resources), Geozentrum Hannover, Hannover, Germany; 7 NOAA Office for Coastal Management, University of New Hampshire, Durham, New Hampshire, United States of America; 8 GEOMAR Helmholtz Centre For Ocean Research Kiel, Kiel, Germany; 9 Christian-Albrechts-University Kiel, Institute of Geosciences, Kiel, Germany; 10 Palaeoceanology Unit, Faculty of Geosciences, University of Szczecin, Szczecin, Poland; 11 CSIR-National Institute of Oceanography, Dona Paula, Goa, India; 12 NIOZ Royal Netherlands Institute for Sea Research, Department of Estuarine and Delta Systems, and Utrecht University, Yerseke, The Netherlands; 13 Ocean and Earth Science, University of Southampton, National Oceanography Centre Southampton, European Way, Southampton, United Kingdom; 14 National Institute of Water & Atmospheric Research, Wellington, New Zealand; Auckland University of Technology, NEW ZEALAND

## Abstract

Commercial-scale mining for polymetallic nodules could have a major impact on the deep-sea environment, but the effects of these mining activities on deep-sea ecosystems are very poorly known. The first commercial test mining for polymetallic nodules was carried out in 1970. Since then a number of small-scale commercial test mining or scientific disturbance studies have been carried out. Here we evaluate changes in faunal densities and diversity of benthic communities measured in response to these 11 simulated or test nodule mining disturbances using meta-analysis techniques. We find that impacts are often severe immediately after mining, with major negative changes in density and diversity of most groups occurring. However, in some cases, the mobile fauna and small-sized fauna experienced less negative impacts over the longer term. At seven sites in the Pacific, multiple surveys assessed recovery in fauna over periods of up to 26 years. Almost all studies show some recovery in faunal density and diversity for meiofauna and mobile megafauna, often within one year. However, very few faunal groups return to baseline or control conditions after two decades. The effects of polymetallic nodule mining are likely to be long term. Our analyses show considerable negative biological effects of seafloor nodule mining, even at the small scale of test mining experiments, although there is variation in sensitivity amongst organisms of different sizes and functional groups, which have important implications for ecosystem responses. Unfortunately, many past studies have limitations that reduce their effectiveness in determining responses. We provide recommendations to improve future mining impact test studies. Further research to assess the effects of test-mining activities will inform ways to improve mining practices and guide effective environmental management of mining activities.

## Introduction

There has been a recent upsurge in interest in deep-sea mining. Many new contractors are applying to the International Seabed Authority (ISA) for licences for exploration of polymetallic nodules in the Clarion-Clipperton Zone (CCZ) in the central eastern Pacific, with sixteen exploration contracts already granted. For the scientific community to provide effective guidance on the impacts from mining, it is important to ascertain baseline conditions, define the types of disturbance that will occur and the probable impact of disturbances from mining [1, 2].

The resource potential of polymetallic nodules is relatively well known. Mining for nodules has been evaluated, and even tested for, since the 1960s. However, deep-sea mining for polymetallic nodules is still a nascent industry. There has been no commercial mining and there is no clear consensus on best available mining techniques. As such, it is very difficult to predict the exact nature of disturbance on the seafloor as this is highly dependent on both the technical developments and the regulatory frameworks that underpin them. However, the global scientific community (and some other stakeholders) have a clear role in providing advice on how deep-sea biological systems could be impacted, their level of biological resilience, the repercussions of biological, geological and chemical changes, and the time required for faunal communities to return to a state similar to that found before the mining activity.

An important way to quantify impacts of mining activities on the deep seafloor is to carry out in situ experiments and monitor recovery from actual disturbance events through time. Many abyssal ecosystem processes required for recovery are slow, which is primarily the result of very low food availability [3], low temperatures slowing biological rate processes [4], low faunal abundances [5] and the patchy distribution of low-quality food [6] rather than any specific effect of pressure [7]. As a result, these experiments will necessarily require long-term monitoring to assess ecosystem impacts and recovery. Such benthic impact experiments have been created by scientists trying to mimic the impacts of mining or by industry testing prototype mining vehicles.

This paper tests whether seabed mining simulations had a negative effect on survival and diversity of meio-, macro- and megafauna. In addition, time-series studies are assessed to evaluate recovery of standing stock and diversity in these groups. This paper assembles all available data into one publication and uses, for the first time, a meta-analysis approach to compare studies and to quantify variation in biological responses to mining activities. We also present the data in formats suitable for planning the next phase of scientific assessments on the consequences of mining. We believe this information will be important for many stakeholders, such as policy makers, regulators and contractors, as the next disturbance “experiments” on the deep seafloor are likely be commercial (test) mining activities.

### Manganese nodule mining techniques

Initial evaluations of deep-sea mining technologies suggested five fundamental engineering approaches: 1) the continuous line bucket, 2) the autonomous shuttle, 3) wireline basket dredging, 4) containers in a pipe and 5) hydraulic dredging [8]. Only three of these technologies have been pursued in practice: continuous-line-bucket-dredge (CLB), wireline basket dredging, and hydraulic dredging. CLB systems use a string of buckets to scoop up surficial sediments [9] and nodules with a maximum penetration depth of around 200 mm. There have been a variety of early tests with CLB technology in the central Pacific in 1971 and 1972 [10, 11] and off Japan in 1975 and 1987 [12]. CLB technology appears unlikely to be adopted by industry and is not further discussed. Wireline basket dredging has been carried out since the first nodules were discovered by H.M.S. *Challenger* [13] and is similar to many biological sampling trawls. However, it is not likely to be scalable to the larger economic recovery of large volumes of nodules. Therefore, this review will focus on what appears to be the most effective system for commercial mining, hydraulic dredging. The specific type of seabed mining equipment that will be used is uncertain, since no mining systems have ever been operated for more than a few days in the deep sea under actual mining conditions. Given our understanding of existing seabed mining technology, seabed mining equipment will most likely consist of a vehicle carrying a collector, which is either on sled runners self-propelled at a speed of about 0.5 m/s, possibly using tank-like tracks [14, 15] or Archimedes screws that disturb the sediment in two wide tracks [16]. The collector would likely be at least 6 m wide (current discussions range up to 14 m) and would collect nodules in surface sediments by mechanical means or separated from the sediment using water jets (hydraulic) [14, 15]. The collecting devices make a first separation of the nodules from the surrounding sediment using water jets, rake tines and comb teeth [17]. They are also designed to have a controlled digging depth into sediments as the nodules are primarily located in the upper 10 cm of the sediment [17]. The seabed collecting devices would be connected with hydraulic (or air-lift) pumping systems that pump the nodules from the seabed to the surface through a riser system [15, 18]. During mining operations, some of the flocculent surficial sediment would be resuspended by hydraulic jets and movements of the mining collector. Deeper sediment layers may be broken up into lumps that could partly enter the collection system [14]. The vehicle is likely to compact the underlying sediment. Behind the vehicle an unevenly disturbed field would persist in the track areas (Fig 5 in [15]). Resuspended sediment plumes would settle on both over the disturbed area and surroundings [15]. The residual sediment carried to the sea surface with the nodules would likely be separated from the nodules and discharged near the seabed. The resedimentation of material from multiple sediment plumes (i.e., created by collector and from deep-sea discharge of lifted sediments) has the potential to impact much larger seafloor areas than directly impacted by removal of the nodules themselves [19].

For effective mining, the seabed collector vehicle will likely follow a ‘lawnmower pattern’, moving back and forth along roughly parallel tracks, leaving only small remnant unmined area with high-value nodule patches [14]. However, nodule fields themselves are patchily distributed on 0.1–10 km scales [20], often following the ridge and valley topography characteristic of the CCZ [21], suggesting that areas of minable nodules will be separated by swathes of low-value sediments of order 0.1 to 10 km wide. This suggests that within a typical mining area, covering between 10 and >100 km^2^, nodule-rich patches would be nearly totally disturbed, while intervening unmined swathes potentially much greater in area would be impacted by sediment plumes. An exploration license, for example those issued by the ISA, can cover areas (not necessarily contiguous) of 75,000 km^2^ [22]. Early assessments of most exploration licenses suggest that 20 to 30% of exploration claims may have suitable nodule resources and are sufficiently flat for mining vehicles (usually <2° slope). An area of about 8500 km^2^ is estimated to be sufficient to support about 20 years of polymetallic nodule mining [22], although the area disturbed by mining activities, particularly plumes, is likely to be larger. This broad scale of activities means polymetallic nodule mining could become a pervasive stressor in remote abyssal ecosystems. It could affect many marine organisms, especially those attached to the nodules, and cause profound ecological shifts. Clearly the effects of large scale disturbance of abyssal sediments need to be assessed and quantified.

### Seabed disturbance experiments relevant to polymetallic nodule mining

**Fig 1:**
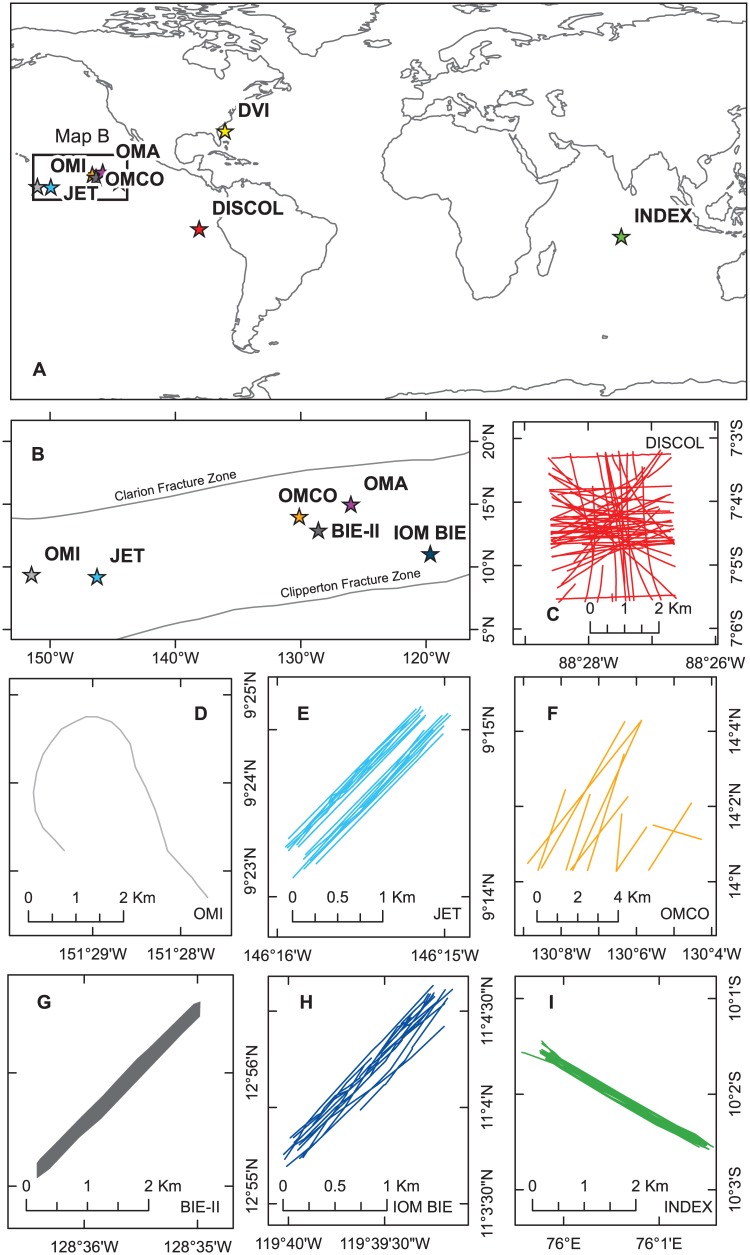
Maps of the locations of deep-sea mining simulations and test mining activities. A) Map of the world with deep-sea mining simulations and test mining activities marked as stars coloured according to the convention used throughout the paper; B) zoomed in map of the Clarion Clipperton Zone (extent indicated on map A); C-I) Maps of individual deep-sea mining simulations and test mining activities: C) DISCOL; D) OMI (DOMES A); E) JET; F) OMCO sled tracks investigated in [16]; G) BIE-II (note that individual tracks not discernible, so map shows polygon of extent of tracks; H) IOM BIE; I) INDEX. Latitude and longitude labels are on the right and base of each map.

The first attempt to mine manganese nodules using airlift pumping was carried out in 1970 by Deepsea Ventures Inc. (DVI) in 800 m deep water on the Blake Plateau, off Florida (Fig 1), in the North Atlantic Ocean [23, 24]. The mining system used a collecting device at the seafloor with airlift pumping to the *Deepsea Miner* surface vessel [8, 23, 25]. Following more extensive commercial resource evaluation the central Pacific emerged as the most likely area for nodule mining activities. In 1976 the first test mining operation in this area [26, 27] was carried out by the Ocean Mining Associates (OMA) consortium using the *Wesser Ore* (an iron ore carrier that was renamed *Deepsea Miner II* in 1977), a suction dredge towed on skis and a solid riser airlift system. In 1978 three further test mining operations took place in the Pacific. The first was carried out by Ocean Management Inc. (OMI) from the vessel M.V. *Sedco 445*. The impacts were studied from the R.V. *Oceanographer* as part of the Deep Ocean Mining Environmental Study (DOMES) project [28]. The test mining, at DOMES site A (labelled OMI on Fig 1), was done in two phases. The first phase (15 March to 14 April 1978) used a hydraulic lift system and the second phase (19 April to 10 May 1978) used an air lift system; the former being more effective. Actual test mining took place during three relatively short periods: March 28 (15 hours), April 6–8 (54 hours) and May 1–4 (33 hours; all 1978). During the mining activities, the nodule collector was towed along the seafloor at the end of a long rigid pipe string with a flexible hose connection to the collector. The collector was supported on skids and collected nodules along a path around 3 meters wide. Most of the sediment was pumped out at the seafloor [28]. When operating optimally, the systems recovered nodules at a rate equivalent to 5 x10^6^ kg per day [29]. Both the surface discharge waters and benthic plumes generated by the test mining were studied [28]. The second test mining in 1978 was completed at DOMES site C (to the east of site A; labelled OMA in Fig 1) by DVI for OMA on 10 November 1978 [30]. This system consisted of a collector device with multiple dredge heads towed on the seafloor. The nodules were pumped via a solid pipe to the surface vessel R.V. *Deepsea Miner II*. This test lasted for about 18 hours and collected around 5.2 x10^5^ kg of nodules [30]. The third of the 1978 tests, also in November [25], was undertaken by the Ocean Minerals Company (OMCO), a consortium of several industry groups led by what is now Lockheed Martin, using the *Hughes Glomar Explorer* ship in the central area of the CCZ (outside areas currently licensed by the ISA) [16, 31–33]. In 1979, the OMCO consortium completed their mining test (as the 1978 test was suspended prior to the mining vehicle reaching the seafloor [34]), which was the last one known to date in the CCZ (Chung, 2009). After the intensive mining activities of the late 1970s, commercial interest in mining for polymetallic nodules declined and the test mining vehicles were mostly scrapped.

In 1988 and 2004, dives of the submersible *Nautile* revealed the presence of tracks on the seafloor in the area of operation of the OMCO consortium [16, 31, 32] (Fig 1). The size and shape of these tracks do not match the size and shape of the nodule collector tested by OMCO. Their position as well as their shape is rather more consistent with dredge sampling carried out by OMCO in 1978 [33].

Although some attention was focussed on mining of metalliferous muds in the Red Sea in the late 1970s [35–38], the next major nodule mining-related disturbance to the deep sea floor was off Peru (Fig 1) in 1989. The *Dis*turbance and Re*col*onization Experiment (DISCOL) in a manganese nodule area of the deep equatorial eastern Pacific Ocean was conducted as part of German national deep-sea environmental protection activities in 1989 [14]. An 11 km^2^ area of seabed was ploughed with a specially designed ‘plough-harrow’ [39] that disturbed the upper layers of sediment (hundreds of mm) and buried the nodules across its 8 m width as it was towed in a 3.7 km diameter circular experimental area (DEA). Seventy eight deployments of the plough-harrow were made in different directions [14]. The sediment suspended by this activity settled out over the DEA in a layer up to 30 mm thick [14]. The DISCOL site was extensively re-surveyed as part of the original programme using a variety of methods. Apart from a baseline survey before the disturbance experiment, further sampling was undertaken 0.5, 3 and 7 years later [14]. More recently the DISCOL area was resurveyed in 2015, twenty six years after the initial disturbance [40, 41], as part of the internationally funded Joint Programming Initiative Healthy and Productive Seas and Oceans (JPIO) project “Ecological Aspects of Deep-Sea Mining”.

Following a review in 1984 of the Ocean Minerals and Energy Division’s (OMED) environmental research related to deep-sea mining, the US National Academy of Sciences recommended that the US National Oceanic and Atmospheric Administration (NOAA) conduct a small-scale experiment to assess the impacts of sediment resuspension and deposition [42]. An initial unsuccessful effort was made with a Remote Underwater Manipulator (RUM3) device in 1990 at the DOMES C site (Fig 1). This was followed by the Benthic Impact Experiment (BIE) in 1991 using the Deep Sea Sediment Re-suspension System mark 1 (Disturber) deployed from a Russian vessel R.V. *Yuzhmorgeologiya* in the CCZ (following initial tests in 4000 m deep water off California). This was also not successful, owing to winch problems. The programme was repeated in 1992, during which 44 tows of the disturber were carried out. Subsequent analyses of sediment trap and core records revealed that the disturber design was not effective at resuspending sediment and so the disturber was redesigned. In 1993 the BIE-II project used the new disturber (Deep-Sea Sediment Resuspension System—DSSRS [43], which completed forty nine successful tows (Fig 1). The DSSRS dredged bottom sediment and resuspended a total of 4,000 m^3^ of wet sediment as a plume above the seafloor. The impacts were evaluated a year later in 1994 [42]. The DSSRS device consists of a towed frame weighing 3.2 tonnes, of dimensions 4.8 m long x 2.4 m wide x 5.0 m tall [43]. A mounting frame on the top of the DSSRS stack allowed deployment of a rosette sampler to provide estimates of sediment discharge into the water column. Additionally, 18 sediment traps and two current meters were arrayed across the study site to assess far field sediment deposition. Prior to and following the DSSRS tows, randomly-located replicate box core and multicore samples were collected to assess simulated mining activity impacts on the deep-sea benthos [44].

In 1991, the Metal Mining Agency of Japan (MMAJ) began a project focussing on environmental research for manganese nodule mining. In 1994, the “Japan Deep-Sea Impact Experiment (JET)” was carried out as part of this project to test the effects of sediment resuspension and redeposition from mining activities [45]. The experiment used the same DSSRS benthic disturber as described above for BIE-II. The disturber was towed over 19 transects in the experimental area (Fig 1), discharging around 350 tons (dry weight) of sediment [45], which reached thicknesses of up to 19.5 mm on the seafloor [46]. A mooring with sediment traps was deployed and a series of multicore samples were taken prior to disturbance. The multicore samples were repeated, seafloor photographs taken and the mooring was recovered shortly after the disturbance [45]. Box core samples, for macrofaunal analysis [47], and towed camera photographs, for megafaunal analysis [48], were obtained on one cruise, 2 years after the disturbance [47].

In July-August 1995, a benthic impact experiment (IOM BIE) was carried out by the InterOceanMetal (IOM) Joint Organization. IOM is an intergovernmental consortium set up for preparation of commercial nodule development in the eastern part of the CCZ. Following preliminary surveys the IOM BIE experiment used the same DSSRS system [49, 50]. In all, 14 tows, each around 2.5 km long, were carried out on a site of 200×2500 m and the impact was observed from deep-sea camera tows and sediment samples [51]. The test site was revisited in April-May 1997, June 2000 [21, 52] and in March 2015 as part of the JPIO project “Ecological Aspects of Deep-Sea Mining” [53].

The Indian Deep-sea Environment Experiment (INDEX) was started in 1995 by the National Institute of Oceanography, Goa, to investigate the impacts of disturbance from nodule mining in the Central Indian Ocean Basin. Once again the DSSRS hydraulic device (as used for JET, IOM BIE and BIE II) was used to simulate mining disturbance in an area of 3000 x 200 m (in a NW-SE orientation) over a period of 9 days during August 1997. The DSSRS resuspended more than 6000 m^3^ of sediment during 42 hours and 14 minutes of operations (the time the sediment resuspension pumps were on) covering a total distance of 88.3 km. Except for the first tow which was about 1 km long, all other tows were the full length of the disturbance strip (> 3 km). The disturbance was monitored before, during and after the disturbance with moorings at 10 locations (with current meters, sediment traps and transmissometers). Four acoustic transponders were also deployed around the disturbance area for accurate positioning of the CTDs, towed cameras, box corers and multicorers that were used to assess the impact of the disturbance [54]. The work at INDEX was carried out during four expeditions onboard RV *Sidorenko* and two onboard RV *Yuzhmorgeologiya* [54, 55].

In 1997, MMAJ (Japan) conducted ocean tests of a towed nodule mining system in 2200 m water depth the vicinity of the Marcus-Wake Seamounts of the North Pacific Ocean. In total, 7.25 tons of nodules were recovered with a collector efficiency estimated at 87% [56]. This site was revisited twice, one month after sea trials to examine the mining tracks with a ROV, and a year later using a towed camera platform. A further experiment at the same site was done in 1999 using a scraper, 6 m wide × 1 m long × 0.4 m tall, which removed and piled nodules up by bulldozing the top surface sediment-nodule layer. The scraper was towed 15 times in a very localised area, 200 m long and 100 m wide [57]. The site was assessed using camera tows and multi-corer samples immediately after the experiment and after one year. Unfortunately, only limited data on the sea trials have been published [57–59].

Various small-scale nodule collection and biological sampling activities have taken place more recently with epibenthic sleds and dredges, which may be useful to assess localised disturbance. These investigations are ongoing, being done as part of routine environmental surveys. In the CCZ such collections have taken place at least in the Belgian Claim area in 2014 (using the M.V. *Mt Mitchell*) and on several recent cruises to the German Claim area: 2010—RV *Sonne* SO205 to the eastern German area [60, 61]; 2012—*L 'Atalante* BioNod ´12 to the French and eastern German area; 2013, 2014, 2016—RV *Kilo Moana* MANGAN 2013, MANGAN 2014 and MANGAN 2016 to the eastern German area; 2015—RV *Sonne* SO239 to the eastern German area, the IOM area, the Belgium and French area [62], to the IOM area (2001, 2004 and 2009) and to the UK area (RV *Melville* 2013 & RV *Thompson* 2015 cruises by UKSRL, ABYSSLINE Project).

## Methods

**Fig 2:**
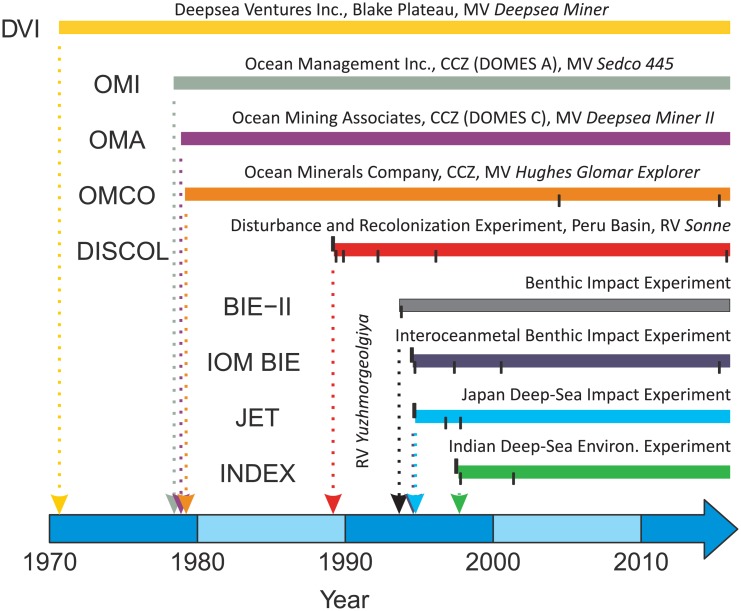
Timeline of deep-water seabed test mining or mining simulations. Bars represent time since initial disturbance to the seafloor. Upward ticks indicate the timing of pre-disturbance visits. Downward ticks indicate the timing of post-mining monitoring visits. Short name indicate in capitals and full name of each experiment indicated above each bar. OMI, OMA, OMCO, BIE-II, IOM BIE and JET experiments were carried out in the Clarion Clipperton Zone (also indicated as CCZ). The INDEX experiment was carried out in the Indian Ocean. Note OMCO disturbance investigated was sledge samples and not the mining vehicle test.

We used a meta-analysis approach to examine the impacts of mining activities in the studies listed above (see Fig 2 for a summary of the timeline). Meta-analysis focuses on the direction and magnitude (represented by effect size) of the consequences of a treatment (in this case simulated mining disturbance) across studies. Use of a standardised measure of “effect size”, the standardised mean difference between control and disturbed samples (see below), allows the studies to be compared directly.

We searched the biological literature for studies that reported the effects of simulated mining disturbance on deep-water marine organisms such as those described above. Literature searches were conducted using the ISI Web of Science Database (using the keywords deep, sea, nodule, mining, impact), following reference lists in papers and through expert consultations. Owing to the relatively small number of studies, we compiled all available literature of any age, including both peer-reviewed studies and “grey literature”. Inclusion of the “grey literature” was particularly important as the results of some mining studies have only been published in conference proceedings. All relevant studies were included in the descriptions of past commercial test mining or scientific disturbance events designed to simulate mining.

**Fig 3:**
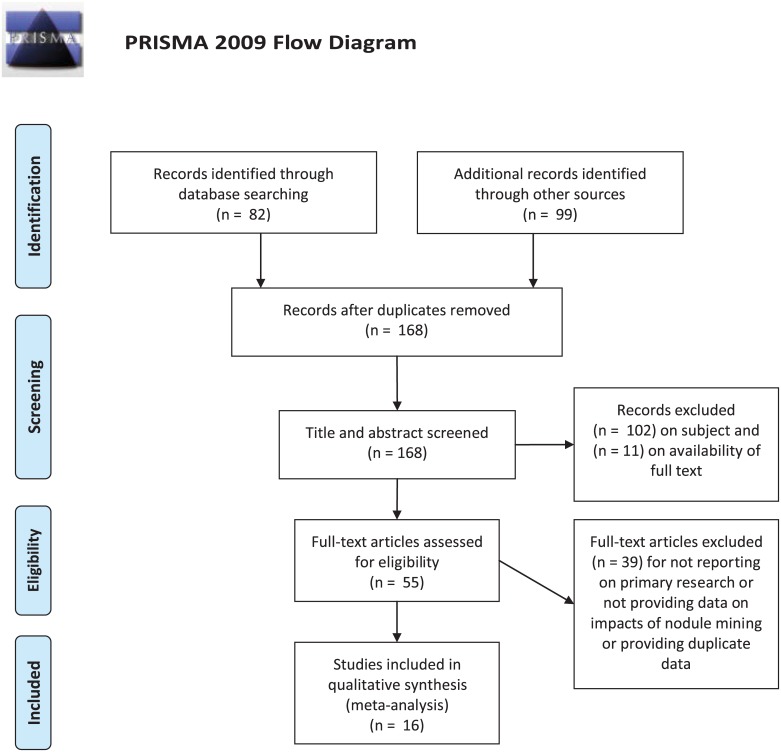
Flowchart of study identification and selection process. All systematic review and meta-analyses methods conducted according to PRISMA guidelines. See PRISMA checklist in S3 Table. *From*: Moher D, Liberati A, Tetzlaff J, Altman DG, The PRISMA Group (2009). *P*referred *R*eporting *I*tems for *S*ystematic Reviews and *M*eta-*A*nalyses: The PRISMA Statement. PLoS Med 6(7): e1000097. doi:10.1371/journal.pmed1000097. **For more information, visit** www.prisma-statement.org.

For the quantitiative analysis, all studies were screened for relevance to the study by reading the title and abstract, 66 studies appeared relevant after this. Full text was obtained for the studies that were selected by the subject-based screening. Obtaining full texts for some of these studies was challenging and 11 studies were not possible to obtain. From careful inspection of the abstracts it appeared that these studies with missing full text were all summaries or repeated data that were available elsewhere. Full texts of the remaining papers were examined and studies excluded if they did not report on primary research, did not contain any data or data suitable for the analysis or provided data duplicated in another paper (Fig 3). A total of 16 studies provided data for the quantitiative analysis.

Data from a total of 12 small-scale commercial test mining or scientific disturbance events designed to simulate mining were available. Of these, five (DVI 1970; OMI 1978; OMA 1978; OMCO 1979; MMAJ) were commercial test mining or exploration activities and the remainder were scientific studies. Of the eight scientific disturbances, all apart from four (DISCOL, INDEX, MMAJ x 2) were in the CCZ in the equatorial eastern Pacific Ocean. The five commercial test mining activities collected nodules, but most of the scientific studies did not. Scientific studies at five sites used the same benthic disturber device (BIE I, BIE II, JET, IOM BIE, INDEX).

The impact of disturbance on benthic fauna was only assessed at seven sites (OMA/DOMES C 1978, OMCO 1978, DISCOL, BIE II, JET, IOM BIE, INDEX). At the DISCOL site a plough-harrow was used to bury nodules and disturb the sediment. With the exception of DOMES C, these sites were generally extensively studied and multiple faunal and physical measurements were made. The sampling design was reasonably robust in most studies: a control (i.e., undisturbed site) was investigated at five sites (OMCO 1979, DISCOL, BIE II, JET, INDEX) and conditions prior to disturbance were assessed at five sites (DISCOL, BIE II, JET, IOM BIE, INDEX). Five sites were investigated more than once after disturbance (DISCOL, BIE II, JET, IOM BIE, INDEX) providing a time series to assess recovery (Table 1).

**Table 1:** Summary table of previous deep-water disturbance studies relevant to mining (see Fig 1 for a map and Fig 2 for a timeline of these studies).

Site	Revisits	Pre-data	Levels	Investigations	References
OMCO	26y	No	In/out track	Meio	[16, 31]
OMA (DOMES C)	5 (failed), 12y	No	In/out track	Macro (not published, low n)	
DISCOL	0, 0.5, 3, 7, 26y	Yes	Low/high/ref	Meio, Macro, Mega	[63–66]
BIE-II	1m, 1y	Yes	In/out track	Meio, Macro	[44]
JET	2w, 2y, 3y	Yes	Light/med/heavy	Meio, Macro, Mega	[47, 48, 67, 68]
IOM BIE	8m, 2.4y	Yes	In/out track	Meio, Mega	[50]
INDEX	1m, 3.8y	Yes	In/out track	Meio, Macro	[69, 70]

Our analysis included all experiments that reported the mean response, error, and sample size in a control and disturbed treatment. In many cases, data were available for both control sites (i.e., representative undisturbed sites sampled concurrently with the disturbed sites) and pre-disturbance conditions (the site of disturbance sampled prior to disturbance activity). Disturbed treatment sites were preferentially compared with control sites, although comparisons were made with pre-disturbance data if no control sites were investigated. In several cases, if not reported in the literature, we were able to obtain raw data from the original authors of the studies to calculate the parameters. Data were preferentially obtained from tables and raw data. However, in some cases data were digitised from graphs using ImageJ (v1.44 National Institutes of Health) software. In addition, we obtained as much metadata as possible (S1 Table) for each study. A table of all known disturbance studies and cruises to investigate them was compiled to guide future studies (S2 Table).

A range of faunal groups have been recorded during the disturbance experiments. The meiofauna (a faunal size fraction typically passing through a 500 μm—1 mm sieve and retained on a 32–63 μm sieve; see S1 Table) have been fairly intensively studied, with records of Foraminifera [67], Nematoda [16, 31, 50, 66, 68, 69], Harpacticoida [31, 50, 66, 68, 69], Ostracoda [31, 66], Polychaeta [66, 69], Halacaridae [31], Tardigrada [31], Kinorhyncha [31, 69], Mollusca [31], Rotifera [31], Nemertea [69], Platyhelminths (Turbellaria) [69], Gastrotricha [69] and early life-stages of larger fauna [66]. The macrofauna (a faunal size fraction typically retained on a 300–500 μm sieve; see S1 Table) have also received some attention, with studies assessing Polychaeta [44, 47, 55, 63], Arthropoda [44, 47, 55, 63], Mollusca [44, 63] and Echinodermata [63]. Megafaunal assessments, focussing on the entire faunal assemblage (identifiable in imagery), have been carried out at three sites [48, 65, 71].

Many studies included assessment of more than one species or faunal group (e.g. megafauna and macrofauna) in a given experiment. If the responses of multiple species or faunal groups were tested in the same experiment, the responses of all species and faunal groups were included. Although this could decrease the independence of some data points, it allowed us to explore responses across a broader range of taxa. If the experiment reported the response over time, all time points were recorded and either used as a time series, or specific response periods (e.g. responses within 1 year) were used in the analyses. Two types of response variable were assessed: density and diversity. If an experiment reported more than one aspect of diversity, measures were selected that were most comparable across studies. Most studies focussed on overall assemblage diversity, rather than diversity of specific taxa. If multiple sediment depth horizons were assessed separately in a study, these were combined to give a total value.

### Data limitations

Almost every investigation of disturbance has used a different sampler size (e.g. sediment cores of various sizes) and sieve-size combination (S1 Table). A range of sieve sizes have been used for meiofaunal assessment (32, 40, 45 and 63 μm). All macrofaunal samples in the data assessed were sieved through a 500 μm sieve, except JET, where a 300μm sieve was used. Meiofauna have been enumerated from sediment samples of 6.2–2000 cm^2^ and macrofauna from 50–2500 cm^2^. Almost all studies were internally consistent in methods throughout the time series. However, in the INDEX study, a different sediment subsample size was used in assessments of recovery [55] from that used during the initial assessments of disturbance [70]. Standardising the differences between control and experimental treatment improves the quality of comparisons, but caution should still be applied in generalising results from different sizes and sampling effort, particularly in comparing diversity measures, which typically do not scale linearly with sample area [72].

It appears that there are several reported and unreported limitations in the data available. One primary limitation is in the accuracy of location. Many of the studies, particularly the older ones, relied on imprecise navigation and relocating disturbed areas was not always reliable. As a result, disturbed areas may have been missed or the sample might have been inadvertently misassigned. Other methodological issues within studies have also been identified. In the DISCOL megafaunal studies, photographs were in parts taken selectively by an operator (rather than at a continuous interval). This would lead to a general positive bias in the results (i.e. no photos without organisms). Furthermore, the bias may change throughout the survey towards more charismatic or less common individuals. The influences of these factors is not possible to remove and difficult to evaluate.

The abyssal deep sea typically has low densities of fauna, particularly of larger size classes [73], although that is not always the case [20]. Furthermore, diversity is high [5, 74, 75] and many species are represented as singletons in small sediment samples. As a result, large samples are required to quantify faunal density and especially diversity. Even whole box-core samples (~0.25 m^2^) require numerous replicates to properly characterise macrofaunal communities (with individual densities typically <200 specimens per box core). It is clear that undersampling of fauna is an issue with the studies investigated here, with 59% of density measurements in control sites (for all size classes) having a mean of <30 individuals per sample (28% having <5 individuals). These problems are greater in macrofaunal samples, but are still present in meiofaunal samples (84% and 39% of control samples have a mean number of <30 individuals per sample for macrofauna and meiofauna, respectively). These problems are often hidden in density measurements if they are standardised to a larger area or volume. Low faunal numbers make detection of impacts difficult by reducing statistical power. No studies were removed from the analysis as a result of low faunal numbers, but all studies where the total numbers of organisms counted in the control site was < 30 are marked so they can be identified as potentially less sensitive to detecting disturbance effects. Very small sub-sample areas were used for macrofaunal assessments during INDEX (50 cm^2^ [70] and 113 cm^2^ [55]), which contained a maximum number in any sub-sample of only 12 individuals and most samples had considerably fewer [55].

Replication is reasonably high in most studies included here, and the overall mean number of replicates at control sites was 9.8, and 7.5 at disturbed sites. Pseudo-replication occurred in the INDEX study, where three sub-samples from each box core were treated as true replicates. As a result of the limitations, the INDEX data should be treated with caution particularly.

### Data analysis

Meta-analysis techniques enable us to assess standardised differences between control and impacted samples (Eq 1, corrected following Eq 5) and their confidence intervals (variance calculated using Eq 3, corrected using Eq 6, and then converted to confidence intervals using Eq 15). This enables calculation of a weighted mean of all studies (Eq 13) with associated confidence interval (variance calculated using Eq 14, and converted to confidence intervals using Eq 15).

The first step in the analysis was to compute the treatment effect size. As the studies were often reported on different scales and used different methods to collect and analyse data, the standardized mean difference (SMD; also referred to as Cohen’s d) between the control and impacted treatments was assessed [76]. This divides the difference between the control and treatment by that study’s standard deviation to create an index that is comparable across studies [77]. The SMD was calculated as Cohen’s d (Eq 1), where $\bar{X_{1}}$ and $\bar{X_{2}}$ are the sample means of the two groups and *S*_*pooled*_ is the pooled standard deviation, calculated using Eq 2. Although the underlying population standard deviation should be the same in the two sample estimates, a more accurate estimate is obtained by pooling the sample standard deviations.

$$d=\frac{\bar{X_{1}}-\bar{X_{2}}}{S_{pooled}}$$ (1)

$$S_{pooled}=\sqrt{\frac{\left(n_{1}-1\right)S_{1}^{2}+S_{2}^{2}\left(n_{2}-1\right)}{n_{1}+n_{2}-2}}$$ (2)

The variance of *d* is approximated by Eq 3. The standard error of *d* is the square root of *V*_*d*_:

$$V_{d}=\left(\frac{n_{1}+n_{2}}{n_{1}n_{2}}\right)+\frac{d^{2}}{2\left(n_{1}+n_{2}\right)}$$ (3)

The sample sizes in mining studies are often small, which can lead to a bias on the high side in SMD. As a result we used a correction factor J (Eq 4) to transform the SMD and variance from *d* to Hedge’s *g* (Eq 5), using the following small sample size bias correction, where *df* is the degree of freedom used to estimate S_pooled_, which for two independent groups is *n*_*1*_+*n*_*2*_-2:

$$J=1-\frac{3}{4df-1}$$ (4)

g=J×d (5)

$$V_{g}=J^{2}\times V_{d}$$ (6)

To summarise the results of the studies a random-effects meta-analysis was used, as we expect that the true effect would vary between studies because of factors including different disturbance methods and different biological communities. In order to account for the differing sample sizes of the individual studies, we weighted the estimates from each study. The between-study variance (*T*^*2*^) is first calculated, where *k* is the number of studies, W is the inverse of the variance (1 / V_g_) and Y = g:

$$T^{2}=\frac{Q-df}{C}$$ (7)

$$Q=\overset{k}{\underset{i=1}{\sum }}W_{i}Y_{i}^{2}-\frac{{\left(\sum_{i=1}^{k}W_{i}Y_{i}\right)}^{2}}{\sum_{i=1}^{k}W_{i}}$$ (8)

df=k−1 (9)

$$C=\sum W_{i}-\frac{\sum W_{i}^{2}}{\sum W_{i}}$$ (10)

The weight assigned to each study is:

$$W_{i}^{*}=\frac{1}{V_{y_{i}}^{*}}$$ (11)

$V_{y_{i}}^{*}$ is the within-study variance for study I plus the between-studies variance T^2^

$$V_{y_{i}}^{*}=V_{y_{i}}+T^{2}$$ (12)

The weighted mean M* is then computed as:

$$M^{*}=\frac{\sum_{i=1}^{k}W_{i}^{*}Y_{i}}{\sum_{i=1}^{k}W_{i}^{*}}$$ (13)

The variance of the summary effect is estimated as the reciprocal of the sum of the weights

$$V_{M^{*}}=\frac{1}{\sum_{i=1}^{k}W_{i}^{*}}$$ (14)

The 95% confidence intervals are calculated from the corrected variance (Eqs 6 and 14)

$$95\%\;confidence\;interval=1.96*\sqrt{variance}$$ (15)

## Results

**Fig 4:**
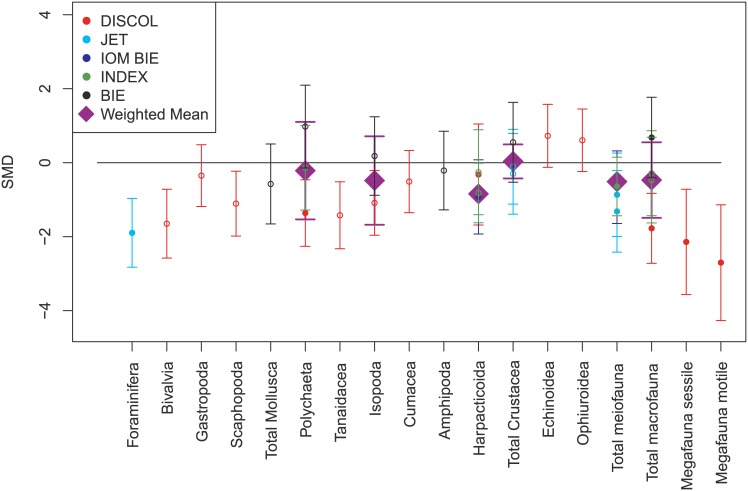
Initial impacts (first repeat visit and less than 1 year after disturbance) of mining activity on densities of a variety of faunal groups. Values represent standardised mean differences (SMD) between faunal densities at impacted sites and control sites and 95% confidence intervals. The horizontal line shows no difference between impacted and control sites. Colours represent different studies. Please note that the disturbances at DISCOL used a different disturbance mechanism than at the other sites. Filled symbols represent more robust data (>30 individuals per sample). Purple diamonds represent weighted means.

**Fig 5:**
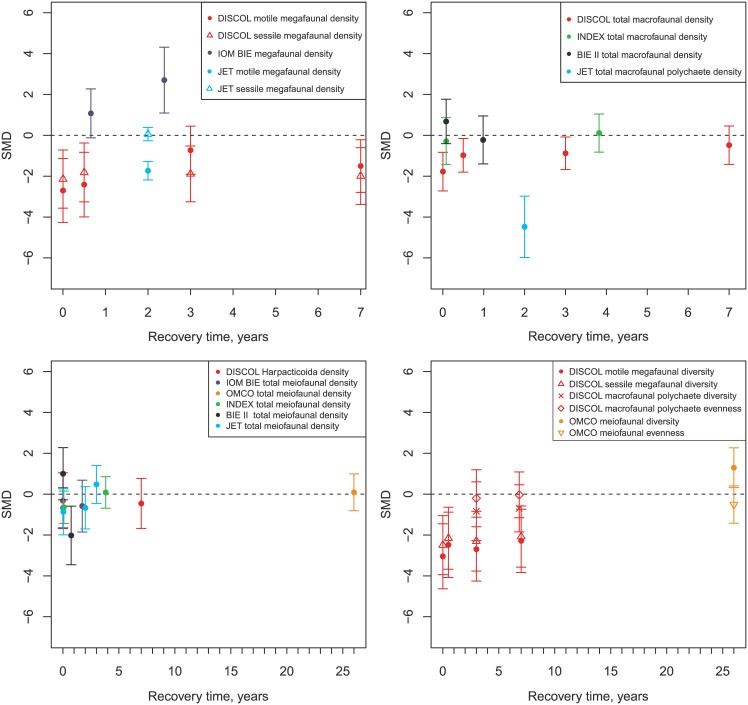
Changes in effects of mining activities over time on faunal density and diversity. Changes shown for megafaunal density (top left), macrofaunal density (top right) and meiofaunal density (bottom left) and diversity (including evenness) of megafauna and meiofauna (bottom right). If totals were not available, the value for the most abundant taxon was plotted and indicated in the legend. Values represent standardised mean differences (SMD) between faunal densities or diversities at impacted sites and control sites and 95% confidence intervals. Diversity was reported as Shannon-Wiener diversity and evenness was Pielou evenness index in the studies used.

The effects of the simulated mining disturbance (Fig 4) are negative for most studies and most groups, and for both density and diversity (H´ and evenness) relative to the controls (Fig 5). The greatest standardised reduction in density following initial disturbance from mining simulations was for polychaete macrofauna at JET. Macrofauna as a group also experienced reductions in the DISCOL and INDEX experiments, although the response of the INDEX macrofauna living deeper in the sediments was minimal (standard deviations included zero) and the data were based on very low faunal densities (Fig 4). Foraminifera showed major reductions in density in the JET experiment (Fig 4). The initial responses to disturbance to diversity metrics were only assessed at DISCOL, where significantly negative changes in both motile (SMD = -3.3) and sessile (SMD = -2.7) megafaunal species richness were observed immediately after disturbance.

There are some exceptions to the general pattern of reductions in density after simulated mining disturbance. At the BIE-II site there are two macrofaunal groups that show an increase in density: polychaetes and isopods, which lead to associated increases in the total crustaceans (the isopods represent 44% of the total crustaceans at control sites) and total macrofauna (isopods and polychaetes represent 24% and 52% of the total at control sites, respectively). These are relatively minor differences. The isopods have low densities in the samples taken before and after disturbance, of <10 individuals in 0.25m^-2^ sample, but the polychaetes are relatively numerous (mean ranges from 28–35 individuals in 0.25m^-2^ sample). In addition, macrofaunal-sized echinoids and ophiuroids increased in overall density after disturbance at DISCOL. When the DISCOL data are examined in detail, all of the positive changes (i.e. SMD > 0) with respect to the control sites are in groups with extremely low densities (<1 individual in 0.25m^-2^ sample). At the JET site crustacean macrofauna and mobile megafauna had slightly greater densities in the disturbed area than outside two years after the disturbance.

When the time series of biological effects of disturbance experiments are considered, there is evidence of minor recovery of density in some groups in some experiments (Fig 5). In the IOM BIE experiments, megafaunal densities were higher than control sites throughout the post-disturbance period and increased between 240 and 870 days after disturbance. In contrast, motile megafauna at DISCOL were reduced in density compared to controls at all time periods, but they appeared to increase in density with time after disturbance. Despite this, significant increases from initial conditions were only observed after three years and, perhaps paradoxically, not after seven years. At DISCOL, total macrofaunal densities were all reduced compared to control conditions, but appeared to increase over time continually, with the largest increases observed between 0.5 and 1 year. At the INDEX site, macrofaunal numbers were not significantly different from control samples in either of the post impact samples (30 days and 1395 days), but total numbers of individuals were very low (<6 individuals per sample). INDEX meiofauna increased in density over time, becoming similar to control conditions by 1395 days. JET meiofaunal density was significantly reduced compared with controls for the 14 and 730 days visits, but densities increased, becoming greater than control values, by 1095 days. The total meiofaunal density at IOM BIE was reduced compared to controls and did not change significantly between 240 and 870 days after disturbance.

Some faunal groups showed no evidence of recovery (Fig 5). The only study of sessile fauna specifically was DISCOL, and sessile megafauna did not show any evidence of recovery. The total macrofaunal density at BIE-II decreased greatly between 1 month and 1 year after disturbance. When the data were examined further, this change was driven by reductions in both polychaetes and crustaceans in the second survey (1 year after disturbance). Densities were virtually identical at the control sites during both time periods and so this decrease in density over year 1 appears robust. Meiofaunal nematodes also showed increased densities over time at BIE-II in the raw data. There were large reductions in nematode densities in both the control and experimental sites between the two surveys (immediately after and 1 year after disturbance), but this reduction was greater inside the disturbed tracks. At DISCOL a major change in meiofaunal communities occurred that appeared unrelated to mining. There were >3x more harpacticoids in undisturbed areas in year 1 than in year 7. The densities of harpacticoids increased over time at the disturbed sites, but not as much as at the reference sites, hence reducing the standardised difference. The differences at INDEX are based on relatively small and variable samples (undisturbed: 0–50 meiofaunal individuals per sample) and have relatively low weights in the analysis.

Changes in diversity could only be evaluated at two sites (DISCOL and OMCO) in five faunal groups (motile and sessile megafauna and macrofaunal polychaetes at DISCOL; total meiofauna and meiofaunal nematodes at OMCO; see Fig 5). It should be noted that the OMCO study did not assess diversity prior to disturbance. At DISCOL the macrofaunal polychaetes had fewer species per sample, lower evenness and lower overall diversity in previously disturbed sites when compared to control conditions. All three indices became more similar to control levels over time, although diversity and richness were still significantly lower after seven years. At DISCOL, both sessile and motile megafaunal species richness was reduced in disturbed areas compared with undisturbed controls (Fig 5). Raw data shows that the species richness of both groups remained approximately similar relative to controls throughout the 7 years of the experiment. At the OMCO site, meiofaunal evenness was lower than background levels within the disturber track (Fig 5), but overall diversity (H´) was higher owing to greater numbers of genera being found in the tracks. However, the assessment of nematodes alone from the same samples shows that all diversity measures at the species, genus and family level are low in samples within the 26 year-old track when compared to samples taken outside the track.

Changes in body size were only evaluated in one experiment (OMCO nematodes). This assessment showed slightly higher body volumes of nematodes within disturbed tracks compared to those outside of the tracks, although these differences were not significant.

## Discussion

All disturbance experiments led to direct physical impacts on the seafloor sediments and removal of seafloor nodules, either through extraction or burial. All experiments also resulted in some level of resedimentation. From seabed photographs alone [78], it is clear that there was variation in the impacts of the different devices, which could have partly contributed to the differences in recovery seen between the studies. For example, of all the sites that could be analysed for impact-related biological changes, only the disturber at the OMCO site removed nodules, whilst the other experiments typically buried (DSSRS benthic disturber) or laterally redistributed them (plough harrow). There was also variation in the depth of sediment disturbance, with the ploughing system used at DISCOL appearing to be the deepest, disturbing sediments to approximately 150 mm depth [18]. The DSSRS used in the BIE-II, JET, IOM BIE and INDEX experiments was specifically designed to create a plume, with an increase in suspended particles of 300% (from 49 to 150 mg m^2^ day^-1^) observed during disturbance [79]. This value is an average from 10 sediment traps deployed at 7 m above the seabed around the disturber site [79], so it is likely that maximum sedimentation rates would be considerably higher (the highest observed was 244 mg suspended particles m^2^ day^-1^). The effects of this plume were observed to a maximum distance of 250 m from the tracks [79], although the accuracy of these measurements is uncertain. It is not clear how this compared to other experiments, such as DISCOL, where plume effects were not measured, though more recent unpublished studies suggest that burial effects may only have occurred tens of meters from the tracks [41]. Comparable plume model results for BIE-II and DISCOL suggest a similar extent of disturbance for both experiments, with coverage of seabed by > 100 g m^-2^ of sediments predicted to extend approximately 1–2 km from the disturber tracks [80, 81]. The model predictions using representative particle size distributions suggest a wider distribution of sediments than those seen in direct observations [15, 80]. The characteristics of the different disturber systems [82] might also affect changes in the geochemical milieu of the surface sediments [18].

Natural temporal variability in environmental conditions in abyssal areas is high [5, 83, 84] and observed in both control and treatment samples of several experiments. During the IOM BIE post-disturbance survey, massive deposits of fresh phytodetritus were observed in some cores, coinciding with an increase in meiofaunal and megafaunal densities compared to the pre-disturbance baseline [50, 52, 85, 86]. Similarly, during the DISCOL post-disturbance survey, evidence of a pulse of phytodetritus was found in February 1992 [63], which may have contributed to the apparent increase in meiofaunal [66], macrofaunal [64] and megafaunal [65] densities. Obtaining good estimates of natural variance over time is thus necessary to enable natural and human-induced effects to be separated, and hence enable a robust evaluation of the impacts of disturbance.

Of all the faunal groups studied, 64% of the faunal classes, plus grouped meiofauna and megafauna, showed negative impacts in faunal density relative to the controls < 1 year after disturbance. Reductions in density were also observed for polychaetes (INDEX, DISCOL), crustaceans (JET) and total macrofauna (INDEX, DISCOL). Negative impacts of disturbance are observed in most other assessments of similar disturbance in the deep sea, including natural disturbances such as iceberg ploughing [87], the effects of turbidites [88], anthropogenic disturbance such as fishing [89] and oil and gas drilling [90, 91]. These community-level effects result from a suite of biological responses—including movement by motile organisms, predation, mortality and reproduction [92]—and physical actions, such as smothering, burial and compaction [93, 94]. Unfortunately, we know little about the specific effects of these drivers in deep-sea ecosystems, especially in areas with low sedimentation rates, such as the abyssal plains with polymetallic nodules. Several potential ecological phenomena observed in shallower water or terrestrial environments may occur, including spatial (or temporal) intermediate disturbance diversity (or density) maxima [95] and changes to regional diversity (γ- diversity) created through a patchwork mosaic of habitats at varying degrees of disturbance [96]. Although evidence of the impact of these ecological processes is not clearly provided by the mining simulations reviewed, temporal succession in abyssal ecosystems impacted by seabed mining may take hundreds to thousands of years, depending on the scale of the mining impact, because of the slower rates of recolonization observed in the deep sea [97, 98].

The data presented here suggest that some signs of recovery were able to be observed, i.e. there is a general reversion, mainly in density, towards control levels over time, most obvious for meiofauna (but see [16] who studied a compacted track). However, in most cases sites are still significantly depauperate in most faunal groups assessed over decadal time-scales. Succession patterns in the recovery process of benthic communities may involve peaks in the abundance of opportunistic species that benefit from competition release [99].

Species diversity is often more sensitive to change than density [96] and appears to be more significantly impacted, which is also shown here. Recolonisation of benthic communities has long been thought to be slow in the deep sea [97], although recolonisation of deep-sea soft sediment by macrofauna [96] and meiofauna [100] can take place relatively rapidly (months to years). As the experiments reviewed here removed nodules, this could lead to slower recolonisation rates [53], although almost all of the experiments (with the exception of megafaunal evaluations) focussed on the soft sediment fauna and not the fauna associated with nodules. The latter would be unlikely to recover for millennia owing to lack of nodule habitat to recolonize, as the growth rate of new nodules is only a few mm per million years particularly in the CCZ [101].

It is our view that insufficient information is currently available to generalise the observed biological effects to the longer terms, larger scales, and greater disturbance intensities (e.g., from sediment plumes) expected to result from full-scale mining activities [19]. The experiments that have been carried out are few in number and have been confounded by major differences in methodologies, particularly in the nature of the impact. In addition, the spatial scales of disturbance (up to tens of square kilometres), and the intensity and duration (a few days) of plume impacts in these experiments are orders of magnitude smaller than will very likely occur for actual mining. Recolonisation of seafloor communities clearly is scale dependent [102], such that recolonization of vast mined areas of seafloor impacted repeatedly by sediment plumes will require much greater time scales than recovery of the relatively small experimental disturbances reviewed here. In addition, baseline data on the abyssal ecosystems impacted is generally lacking, particularly with regard to ecosystem processes and functions, leading to difficulties in interpreting change. This is coupled with lack of systematic monitoring of experiments and baseline conditions at high resolution over relevant temporal and spatial scales, limiting the power of detecting changes resulting from simulated mining. In essence, the results of the simulated mining studies reviewed here set a lower bound on the likely intensity of mining disturbance effects and the time scales required for benthic community recovery.

It is very important to critically evaluate the findings of disturbance studies, as they play an important role in determining the societal response to, and acceptance of, deep-sea mining. There is a clear need to improve and standardise studies for a better assessment of the effects of large-scale disturbance and for comparisons of the scale and intensity of impacts in different studies. This is particularly pertinent as many contractors to the International Seabed Authority interested in polymetallic nodules are considering test mining at the end of the exploration phase of mining. To understand the impact of any future test mining event, it is necessary to accurately and precisely quantify baseline conditions as well as the nature and extent of the mining impact in space and time. This is challenging and will require careful planning, a multi-disciplinary approach and time-series monitoring of a range of parameters. Furthermore, high spatial accuracy in monitoring samples is required to link these to prior observations of disturbance intensity. A statistically robust sampling plan should be followed and sufficient data are available here to parameterise *a priori* assessments of the amount of sampling necessary to detect an effect of a given size. Variability between samples is observed in the studies here, so it is important to assess multiple sites with sufficient replication to detect effects and distinguish them from background variation. It is clear from the results of this study that sufficient sample numbers and sizes should be obtained to be able to make robust conclusions. This is particularly important for larger-sized fauna, with low population densities, and it is useful to make assessments across multiple size-classes of fauna. To properly characterise the effects of disturbance, it may be necessary to establish an experiment that is large enough to be representatively and accurately sampled over time (probably at least many square kilometres). This may mean that a mining test might be the only practical way to obtain these data. Provision of accessible and quality metadata and data is important to permit comparisons to be made and put into the context of other studies; considerable time was spent in this study identifying and obtaining reference material. Standardisation of equipment and approaches should also be considered to increase the comparisons possible between studies. We have compiled a list of our recommendations and best practices to guide future studies (Table 2).

**Table 2:** Recommendations for robust assessment of the impact of future test mining cases.

Recommendation	Notes
Integrate plan to collect environmental data into plan for test mining	Obtain expert advice in establishing the monitoring aims, design, plan and execution. Plan both spatial and temporal monitoring, considering combined effects, for example from direct mining and redeposition from sediment plumes. Plan to collect multi-disciplinary data using a variety of techniques.
Accurately and precisely quantify the nature and extent of the mining impact in space and time	Understanding the nature of physical and geochemical impacts (e.g., direct community removal, resedimentation, solubilisation of metals) is important for interpreting the effects on biological systems. Data on the temporal and spatial extent and nature of mining impacts allow better links to be made between impacts and effects. Accurate quantification of the impacts experienced by fauna within a specific sample helps guide interpretation of observed effects.
Sampling should follow a predefined sampling design	Sampling should follow a statistically robust sampling design, such as stratified random sampling, which allows truly independent samples to be obtained for analysis. Operator bias should be avoided by following predefined objective criteria for data collection.
Sufficient sample numbers should be obtained	Care needs to be taken to ensure there are sufficient samples to provide the necessary statistical power to detect the effects of mining activities. Statistical power analysis should be carried out prior to sampling to determine the effect size that can be discriminated.
Sufficient sample sizes should be obtained	Faunal densities are low in many mining areas. Therefore, it is vital that a sufficient area of seafloor is sampled to encounter enough organisms for the investigation. For example, at least whole box cores should be used for macrofaunal analysis, and consideration should be taken as to whether larger sampling tools or multiple samples per replicate are required. Megafaunal assessments should cover wide areas. Potentially, for infaunal assessment, focus should be shifted to smaller, more abundant, organisms as these can be captured in large quantities, providing more robust results. Standard sample sizes should be considered to facilitate comparisons. Assessment of multiple size classes of fauna is necessary, because different size classes of organisms may be impacted differently, represent distinct reservoirs of biodiversity and contribute differently to ecosystem functions.
High spatial accuracy in sampling is necessary for reinvestigations of disturbance tracks, and of areas with different sedimentation regimes	Samples should be accurately positioned to properly quantify the impacts of mining. It is important to accurately sample disturbance regimes that have been quantified. It is preferable to be able to direct the sampler itself to land at a planned position, but it is essential to be able to know where it landed with high spatial accuracy (<20m) so that the data collected align with disturbance data. Evaluating disturbed and undisturbed sites in areas where the disturbance itself has limited extension (e.g. tracks of few meter width only) requires video guidance.
Multiple impacted and control sites should be assessed prior to impacts and during all subsequent studies	Mining disturbance in the impacted region should be compared with several control locations. Natural change in the ecosystem may lead to spatially and temporally variable responses in both impacted and control locations. Assessment of multiple sites allows better quantification of variation in the system and hence improves the ability to detect changes and differentiate mining-related change from natural variability. A well formulated and peer-reviewed study design allowing statistically robust analysis should be in place before data acquisition begins.
Methodologies should be standardised to improve comparability between studies	There are multiple methods and processing options for biological studies. Standardisation within a region greatly facilitates meta-analysis. Variables such as sampling volume, method of nodule processing, sieve size, sediment sectioning horizons, photograph altitude, and image resolution offer opportunities for standardisation.
Provide comprehensive metadata and raw data in an accessible way	Future studies depend on being able to quickly revisit sites (to assess recovery) or reanalyse data to make broader comparisons. Without clear metadata (particularly descriptive metadata) and data this is difficult. Providing raw data (pre-processed and post-processed) within a recognised and accessible data repository alongside studies greatly facilitates reanalysis and assessment of long-term changes.

## Supporting information

S1 Table Data used in meta-analysis. See headings tab for more details.
(XLSX)

S2 Table Table of cruises related to simulated mining on the deep seafloor. (XLSX)

S3 Table PRISMA checklist. (DOC)
